# Supplementary material for: Monitoring SARS-CoV-2 seroprevalence over time among pregnant women admitted to delivery units: Suitability for surveillance
Source: PLoS One. 2023 Jan 5;18(1):e0280109. doi: 10.1371/journal.pone.0280109 (PMC9815570; doi:10.1371/journal.pone.0280109)
Supplement: S1 Table — (DOCX) [file pone.0280109.s001.docx]

**S1 table. The STROBE statement- Checklist of items that should be included in reports of cross-sectional studies**

|  | Item No | Recommendation |  |
| --- | --- | --- | --- |
| **Title and abstract** | 1 | (*a*) Indicate the study’s design with a commonly used term in the title or the abstract | The title describes the study as measuring seroprevalence over time. |
|  |  | (*b*) Provide in the abstract an informative and balanced summary of what was done and what was found | Abstract provided. |
| Introduction | | |  |
| Background/rationale | 2 | Explain the scientific background and rationale for the investigation being reported | First five paragraphs of Introduction. |
| Objectives | 3 | State specific objectives, including any prespecified hypotheses | Final paragraph of Introduction. |
| Methods | | |  |
| Study design | 4 | Present key elements of study design early in the paper | “Settings and populations” subsection of Methods. |
| Setting | 5 | Describe the setting, locations, and relevant dates, including periods of recruitment, exposure, follow-up, and data collection | “Settings and populations” subsection of Methods. |
| Participants | 6 | (*a*) Give the eligibility criteria, and the sources and methods of selection of participants | First and second paragraphs of “Settings and populations” subsection of Methods. Consecutive pregnant women of all ages presenting at L&D during daytime excluding weekends. |
| Variables | 7 | Clearly define all outcomes, exposures, predictors, potential confounders, and effect modifiers. Give diagnostic criteria, if applicable | Third and fourth paragraphs of “Settings and populations” subsection of Methods, and “Laboratory methods” subsection of Methods. |
| Data sources/ measurement | 8* | For each variable of interest, give sources of data and details of methods of assessment (measurement). Describe comparability of assessment methods if there is more than one group | “Settings and populations” and “Laboratory methods” subsections of Methods. |
| Bias | 9 | Describe any efforts to address potential sources of bias | All eligible and consenting women were serologically tested and interviewed in the same way. The procedures used resulted in a low proportion declining participation (11%). |
| Study size | 10 | Explain how the study size was arrived at | Sixth paragraph of the discussion. |
| Quantitative variables | 11 | Explain how quantitative variables were handled in the analyses. If applicable, describe which groupings were chosen and why | “Statistical analysis” subsection of methods. |
| Statistical methods | 12 | (*a*) Describe all statistical methods, including those used to control for confounding | “Statistical analysis” subsection of methods. |
|  |  | (*b*) Describe any methods used to examine subgroups and interactions | Symptomatic and asymptomatic women are compared in the subsection “SARS-CoV-2 seroprevalence and risk factors” of the results. |
|  |  | (*c*) Explain how missing data were addressed | “Statistical analysis” subsection of methods. |
|  |  | (*d*) If applicable, describe analytical methods taking account of sampling strategy | “Statistical analysis” subsection of methods. |
|  |  | (*e*) Describe any sensitivity analyses | Supplementary Table 2. |
| Results | | |  |
| Participants | 13* | (a) Report numbers of individuals at each stage of study—eg numbers potentially eligible, examined for eligibility, confirmed eligible, included in the study, completing follow-up, and analysed | “Population characteristics” subsection of Results, and Figure 1 |
|  |  | (b) Give reasons for non-participation at each stage | “Population characteristics” subsection of Results, and Figure 1. |
|  |  | (c) Consider use of a flow diagram | Figure 1. |
| Descriptive data | 14* | (a) Give characteristics of study participants (eg demographic, clinical, social) and information on exposures and potential confounders | Table 1. |
|  |  | (b) Indicate number of participants with missing data for each variable of interest | Table 1. |
| Outcome data | 15* | Report numbers of outcome events or summary measures | Tables 2 and 3. |
| Main results | 16 | (*a*) Give unadjusted estimates and, if applicable, confounder-adjusted estimates and their precision (eg, 95% confidence interval). Make clear which confounders were adjusted for and why they were included | Table 2. |
|  |  | (*b*) Report category boundaries when continuous variables were categorized | Not applicable. |
|  |  | (*c*) If relevant, consider translating estimates of relative risk into absolute risk for a meaningful time period | Not applicable. |
| Other analyses | 17 | Report other analyses done- eg. analyses of subgroups and interactions, and sensitivity analyses | Not applicable. |
| Discussion | | |  |
| Key results | 18 | Summarise key results with reference to study objectives | First two paragraphs of Discussion. |
| Limitations | 19 | Discuss limitations of the study, taking into account sources of potential bias or imprecision. Discuss both direction and magnitude of any potential bias | Final three paragraphs of Discussion. |
| Interpretation | 20 | Give a cautious overall interpretation of results considering objectives, limitations, multiplicity of analyses, results from similar studies, and other relevant evidence | Final paragraph of Discussion. |
| Generalisability | 21 | Discuss the generalisability (external validity) of the study results | Final paragraph of Discussion. |
| Other information | | |  |
| Funding | 22 | Give the source of funding and the role of the funders for the present study and, if applicable, for the original study on which the present article is based | Included in the journal online submission system. |

*Give information separately for exposed and unexposed groups.
