# Supplementary material for: Monitoring SARS-CoV-2 seroprevalence over time among pregnant women admitted to delivery units: Suitability for surveillance
Source: PLoS One. 2023 Jan 5;18(1):e0280109. doi: 10.1371/journal.pone.0280109 (PMC9815570; doi:10.1371/journal.pone.0280109)
Supplement: S2 Table — (DOCX) [file pone.0280109.s002.docx]

**S2 table. Factors associated with SARS-CoV-2 seropositivity by Wondfo.**

|  | **Total**  **N=763** | **SARS-CoV-2 seropositive**  **N=221 (18%)** | **SARS-CoV-2 seronegative N=542** | **OR (95%CI)** | **p-value** |  |
| --- | --- | --- | --- | --- | --- | --- |
| **Age** | | | | | |  |
| <30 years | 409 (53.6) | 72 (52.6) | 337 (53.8) | 1 |  |  |
| ≥30 years | 354 (46.4) | 65 (47.4) | 289 (46.2) | 1.1 (0.7-1.5) | 0.786 |  |
| **Ethnicity** | | | | | |  |
| White | 283 (37.2) | 44 (32.1) | 239 (38.4) | 1 |  |  |
| Non-white | 477 (62.8) | 93 (67.9) | 384 (61.6) | 1.3 (0.9-2) | 0.172 |  |
| **Occupation** | | | | | |  |
| Healthcare | 52 (6.9) | 12 (9.2) | 40 (6.5) | 1 |  |  |
| Essential non-healthcare^a^ | 90 (12) | 17 (13.1) | 73 (11.8) | 0.8 (0.3-1.8) | 0.551 |  |
| Non-essential | 607 (81) | 101 (77.7) | 506 (81.7) | 0.7 (0.3-1.4) | 0.24 |  |
| **Any comorbidity^b^** | | | | | |  |
| No | 613 (80.3) | 115 (83.9) | 498 (79.6) | 1 |  |  |
| Yes | 150 (19.7) | 22 (16.1) | 128 (20.4) | 0.7 (0.4-1.2) | 0.243 |  |
| **BMI** (kg/m²) | 31.3 (5.9) | 31.7 (5.8) | 31.2 (5.9) | 1.01 (0.98-1.05) | 0.389 |  |
| **Height** (cm) | 160.8 (6.5) | 159.2 (5.9) | 161.1 (6.6) | 0.95 (0.92-0.98) | 0.002 |  |
| **Potential COVID-19 symptoms during pregnancy** | | | | | | |
| Fever | 78 (10.2) | 27 (19.7) | 51 (8.1) | 2.8 (1.6-4.6) | <0.001 |  |
| Cough | 134 (17.6) | 43 (31.4) | 91 (14.5) | 2.7 (1.8-4.1) | <0.001 |  |
| Sore throat | 80 (10.5) | 29 (21.2) | 51 (8.1) | 3.0 (1.8-5) | <0.001 |  |
| Myalgia | 98 (12.8) | 43 (31.4) | 55 (8.8) | 4.7 (3.0-7.5) | <0.001 |  |
| Asthenia | 89 (11.7) | 37 (27) | 52 (8.3) | 4.1 (2.5-6.5) | <0.001 |  |
| Coryza | 164 (21.5) | 53 (38.7) | 111 (17.7) | 2.9 (2.0-4.4) | <0.001 |  |
| Anosmia | 86 (11.3) | 45 (32.8) | 41 (6.5) | 7.0 (4.3-11.3) | <0.001 |  |
| Ageusia | 82 (10.7) | 41 (29.9) | 41 (6.5) | 6.1 (3.8-9.9) | <0.001 |  |
| Dyspnea | 72 (9.4) | 28 (20.4) | 44 (7) | 3.4 (2-5.7) | <0.001 |  |
| Headache | 137 (18) | 46 (33.6) | 91 (14.5) | 3.0 (1.9-4.5) | <0.001 |  |
| Fatigue | 82 (10.7) | 34 (24.8) | 48 (7.7) | 4.0 (2.4-6.5) | <0.001 |  |
| No symptoms | 490 (64.2) | 59 (43.1) | 431 (68.8) | 0.3 (0.2-0.5) | <0.001 |  |

Data shown as frequency, n/N (%) or mean (±SD)

BMI = body mass index; CI = confidence interval; OR = odds ratio

^a^ Essential workers defined as those working with food supply or hospital services such as cleaning and hospitality

^b^ Any comorbidity includes diabetes, hypertension, cardiac, lung, rheumatic diseases, cancer and thromboembolic events
